# How Does the Corrected Exhalyzer Software Change the Predictive Value of LCI in Pulmonary Exacerbations in Children with Cystic Fibrosis?

**DOI:** 10.3390/diagnostics13142336

**Published:** 2023-07-11

**Authors:** Irena Wojsyk-Banaszak, Zuzanna Stachowiak, Barbara Więckowska, Marta Andrzejewska, Katarzyna Tąpolska-Jóźwiak, Aleksandra Szczepankiewicz, Paulina Sobkowiak, Anna Bręborowicz

**Affiliations:** 1 Department of Paediatric Pulmonology, Allergy and Clinical Immunology, Poznan University of Medical Sciences, 60-572 Poznan, Poland; marta.andrzejewska.rm@gmail.com (M.A.); ktapolska-jozwiak@ump.edu.pl (K.T.-J.); psobkowiak@ump.edu.pl (P.S.); abreborowicz@ump.edu.pl (A.B.); 2 Molecular and Cell Biology Unit, Department of Paediatric Pulmonology, Allergy and Clinical Immunology, Poznan University of Medical Sciences, 60-572 Poznan, Poland; zuzastachowiak9@gmail.com (Z.S.); alszczep@ump.edu.pl (A.S.); 3 Department of Computer Science and Statistics, Poznan University of Medical Sciences, 60-806 Poznan, Poland; basia@ump.edu.pl

**Keywords:** multiple breath washout, LCI, cystic fibrosis, children

## Abstract

**Aim:** Recently, the most commonly used for multiple breath washout device, the Exhalyzer D, has been shown to overestimate lung clearance index (LCI) results due to a software error. Our study aimed to compare the predictive values of LCI in the CF pulmonary exacerbations (PE) calculated with the updated (3.3.1) and the previous (3.2.1) version of the Spiroware software. **Materials and Methods:** The measurements were performed during 259 visits in CF pediatric patients. We used 39ΔPE pairs (PE preceded by stable visit) and 138ΔS pairs (stable visit preceded by stable visit) to compare the LCI changes during PE. The areas under the receiver operating curves (AUC^ROC^) and odds ratios were calculated based on the differences between ΔPEs and ΔSs. The exacerbation risk was estimated using a logistic regression model with generalized estimating equations (GEE). **Results:** There were statistically significant differences in LCI 2.5% median values measured using the two versions of the software in the stable condition but not during PE. The AUC^ROC^ for changes between the two consecutive visits for LCI did not change significantly using the updated Spiroware software. **Conclusions:** Despite the lower median values, using the recalculated LCI values does not influence the diagnostic accuracy of this parameter in CF PE.

## 1. Introduction

Cystic fibrosis (CF) is an autosomal recessive disorder affecting most organs and body systems including the respiratory system. There are over 160,000 people worldwide living with cystic fibrosis and due to the constantly implemented new standards of care, their numbers are increasing [1]. The median survival rate in the era of highly effective modulator treatment ranges in Europe, Australia, the United States and Canada from 44 to 53 years today [2]. The leading cause of morbidity and mortality in cystic fibrosis are respiratory complications. Despite the advancement in the available treatments and prolonged life expectancy, people living with cystic fibrosis still experience pulmonary exacerbations that negatively influence their quality of life, long-term lung function and survival rates [3]. Therefore, it is of the utmost importance to investigate the potential biomarkers and diagnostic tools that could help in the early diagnosis of pulmonary exacerbations allowing for their expert and timely treatment. Pulmonary function tests are recommended for the diagnosis and monitoring of pulmonary exacerbations in people with cystic fibrosis [4].

Multiple breath washout (MBW) has an established role in cystic fibrosis research and clinical care. The lung clearance index (LCI) derived from MBW is a marker of ventilation inhomogeneity. During the test, while an individual inhales 100% oxygen, the concentration of lung resident nitrogen is calculated. The test is completed when the end-tidal concentration of nitrogen reaches 1/40th of the initial one. The longer the washout takes, the more impaired the homogeneity of ventilation is. LCI itself denominates the ratio of the total amount of air exhaled during the washout (cumulative expired volume–CEV) and the functional residual capacity (FRC) [5].

The lung clearance index has been successfully used for several decades now in children and adolescents as an endpoint in clinical trials [6,7,8,9,10], a marker of lung disease [11,12,13], its progression [14,15,16], and exacerbations [17]. The method is feasible even in preschool children and can be performed by most CF patients. Several studies have been published showing a good correlation of LCI and structural abnormalities in lung HRCT including bronchiectasis and mucus plugging [18]. LCI is especially useful in individuals with mild CF lung disease and FEV_1_ values in spirometry within normal ranges [19].

Recently a sensor-crosstalk error has been described in the commercially available N_2_MBW device (Exhalyzer D, Eco Medics AG, Dürnten, Switzerland) that resulted in the overestimation of LCI results [20]. Subsequently, an updated software (Spiroware 3.3.1, Eco Medics AG, Dürnten, Switzerland) was introduced into the devices that allowed for the recalculation of already existing data. This raised concerns in the CF community about how this error correction would influence the results of the existing clinical studies and the diagnostic value of LCI in CF lung disease [21].

Therefore, we attempted to evaluate whether the utilization of the corrected Spiroware 3.3.1 software influences the diagnostic value of LCI in pulmonary exacerbations calculated with the previously used Spiroware 3.2.1 software (Eco Medics AG, Dürnten, Switzerland).

## 2. Materials and Methods

### 2.1. Study Design

This retrospective study compared the predictive value of LCI measured using the original and updated Exhalyzer D Spiroware software in pulmonary exacerbations in children and adolescents with cystic fibrosis. We analyzed the data collected in our previous study investigating the predictive value of LCI and impulse oscillometry in CF pulmonary exacerbations [22].

In the study, we utilized the measurements performed during consecutive visits of pediatric CF patients (median age 14.5 (6.1–18.0)) treated in the tertiary academic hospital as described before [22]. We included the visits when patients were in stable conditions and visits during pulmonary exacerbations that either resulted in outpatient treatment or hospitalization. We calculated the relative change in LCI absolute values by subtracting the results obtained during the current visit from the results obtained during the previous one. ΔS was calculated if both visits occurred in patients’ stable conditions, and if the pulmonary exacerbation was diagnosed during the visit, we calculated ΔPE by calculating the difference between that and the value at last stable visit.

For the N_2_MBW measurements, we used the Exhalyzer D device with weight-adjusted dead spaces and settings according to ERS/ATS consensus statement guidelines [23]. The data were originally obtained with Spiroware 3.2.1 software and then recalculated with the version Spiroware 3.3.1 introduced after the reports showing inaccuracies in LCI and FRC calculation had been published [20,24]. LCI was calculated as a ratio of the cumulative volume of air expired until the Nitrogen concentration in the expired air equals 1/40 of the original concentration and functional residual capacity (FRC). The higher the LCI value, the less homogenous the lung ventilation. Two other parameters measured during the MBW test are S_cond_ and S_acin_. Scond reflects the convection–dependent inhomogeneity of ventilation and Sacin reflects the diffusion–convection interaction dependent inhomogeneity. Both phenomena occur during the alveolar phase of the expirogram [18].

The Bioethics Committee of Poznan University of Medical Sciences approved the study (no. 386/17). Informed consent was obtained from all subjects involved in the study.

### 2.2. Statistical Analysis

For the assessment of the distribution of data, we used the Shapiro–Wilk test and the F Fisher Snedocor test. The Mann–Whitney test was used for the comparison of LCI values results as well as ΔPEs and ΔSs results without normal distribution.

In order to estimate the risk of pulmonary exacerbation, we used odds ratios (ORs) and the receiver operating curves (ROC). We used the logistic regression model for the calculation of ORs. Repeated measurements in the ROC curves were accounted for using clusters and in the logistic regression model using generalized estimating equations (GEE) [25]. To determine and compare ROC curves, we used the ROC bootstrap procedure for cluster data (STATA, Receiver Operating Characteristic Regression).

Statistical analyses were performed with PQSTAT (Poland) and Stata V16 (StataCorp, College Station, TX, USA) software.

## 3. Results

We collected the data from 251 consecutive visits of 47 patients. In total, 23 patients were female, almost half of them (46.8%) were homozygous for F508del and a further 40.4% carried this mutation on one of the CFTR gene alleles. Six (12.8%) patients were underweight. During the studied period, almost half of the patients (23–48.9%) had abnormal FEV1 (z-score < −1.64) on at least one occasion. The general characteristics of the patients are presented in Table 1.

For the analysis, we used 39 ΔPEs and 138 ΔSs to compare the changes in the LCI in the patients with pulmonary exacerbations. The MBW parameters obtained with both Spiroware software are shown in Table 2. The LCI 2.5% and S_cond_ values during the stable condition obtained with the two pieces of software were statistically different. The difference of LCI 2.5% and S_cond_ during exacerbation as well as S_acin_ in stable condition and during exacerbation did not reach statistical significance.

The median change in LCI parameters measured with the Spiroware 3.2.1 and Spiroware 3.3.1 software is shown in Table 3. For both software, there were statistically significant differences in the changes in LCI absolute values and relative change between two stable visits (ΔS) and exacerbations preceded by stable visits (ΔPE). The differences in S_cond_ were not statistically significant independently of the software used. The only difference between the software was for S_acin_: the difference between ΔS and ΔPE was significant only when Spiroware 3.3.1 was used.

**Figure 1:**
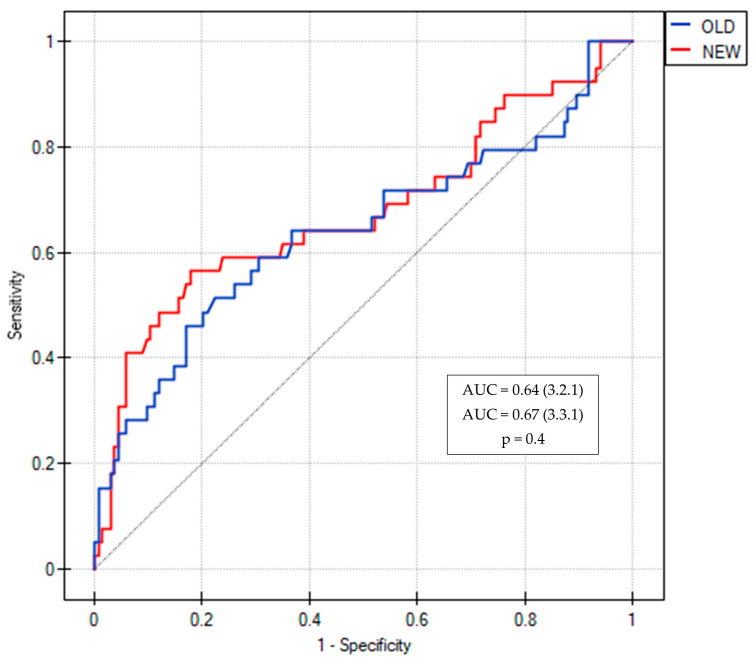
ROC curves of LCI for the prediction of pulmonary exacerbation in CF patients measured with Spiroware 3.2.1 and Spiroware 3.3.1. Abbreviations: OLD—Spiroware 3.2.1; NEW—Spiroware 3.3.1; AUC: Area under the curve.

**Figure 2:**
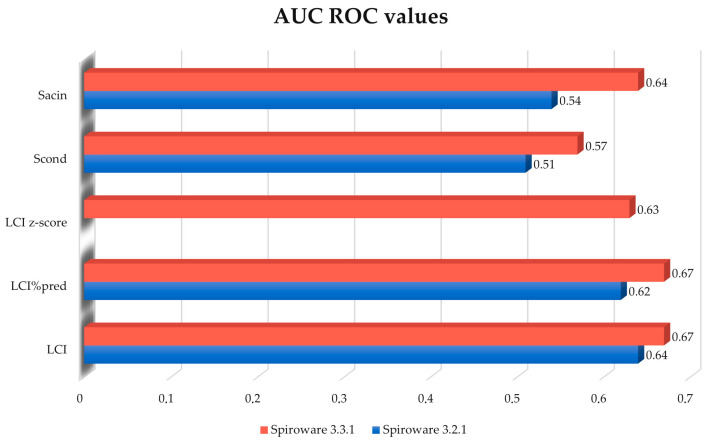
Comparison of AUC of the ROC curves for different MBW parameters for the prediction of pulmonary exacerbation in CF patients measured with Spiroware 3.2.1 and Spiroware 3.3.1. Abbreviations: LCI% predicted—lung clearance index percentage of predicted value; LCI—lung clearance index (absolute value); Scond—index of conductive ventilation heterogeneity (absolute value); Sacin—index of acinar ventilation heterogeneity (absolute value).

The predictive value (AUC^ROC^) of changes between the two consecutive visits for LCI increased from 0.64 to 0.67 using the 3.3.1 Spiroware software but this difference was not statistically significant (*p* = 0.4) (Figure 1). The AUC^ROC^ values for different MBW parameters obtained with both software are shown in Figure 2. The highest AUC^ROC^ values were obtained for LCI absolute values and LCI %predicted that were measured with the Spiroware 3.3.1 software (0.67 both).

**Figure 3:**
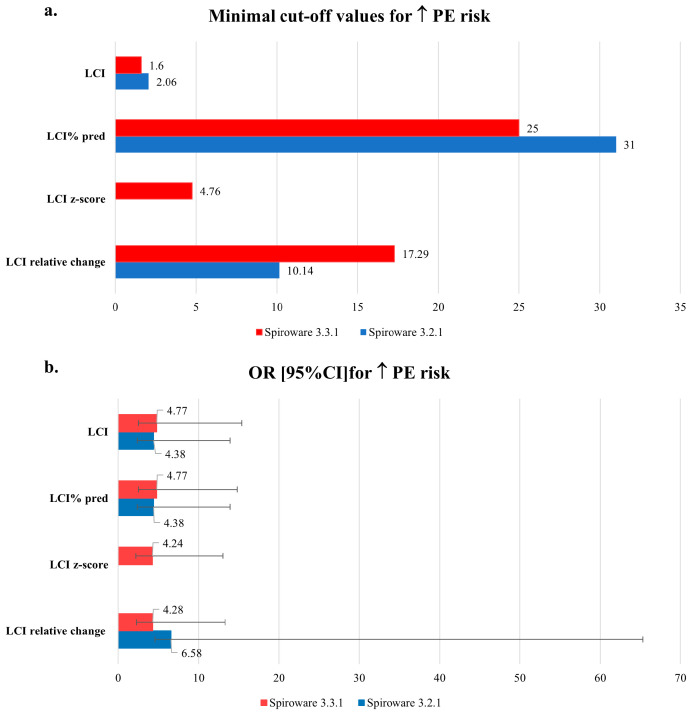
The comparison of: (**a**) minimal cut-off values and (**b**) odds ratios of LCI differences between visits indicating the increased risk of pulmonary exacerbations obtained with Spiroware 3.2.1 and Spiroware 3.3.1 software. Abbreviations: LCI% predicted—lung clearance index percentage of predicted value; LCI—lung clearance index (absolute value); Scond—index of conductive ventilation heterogeneity (absolute value); Sacin—index of acinar ventilation heterogeneity (absolute value).

When Spiroware 3.3.1 software was implemented the cut-off for minimal change in LCI, that was predictive of exacerbation decreased from 2.06 units to 1.6 units with a slightly higher odds ratio: 4.8 vs. 4.4. The cut-off for the relative change in LCI increased, however, from ≥10.1 to ≥17.3. For such a change, the odds ratio for exacerbation was 4.3 vs. 6.6 for the 3.3.1 and 3.2.1 Spiroware software, respectively. The change in LCI % predicted predictive of exacerbation was ≥31 for Spiroware 3.2.1 software and ≥25 for Spiroware 3.3.1 Software. LCI z-score results were only available for the Spiroware 3.3.1 software. We found that the risk of exacerbation increased 4.24 times when z-score values changed by at least 4.76 units. The cut-off values indicating the increased risk of exacerbation as well as its magnitude are shown in Figure 3.

## 4. Discussion

We present here one of the very few studies investigating the impact of improved Exhalyzer D software on the diagnostic value of LCI in pulmonary exacerbations in children and adolescents with cystic fibrosis. We report that despite the differences in LCI values obtained with 3.3.1 Spiroware software, especially in patients in stable condition, the predictive value for pulmonary exacerbations of LCI change between consecutive visits remained very similar.

Following the published reports on the cross-sensitivity error in the Exhalyzer D that resulted in overestimated MBW outcomes and prolonged washout time, a significant amount of concern has been raised as to the reliability of previously published clinical trials utilizing LCI as endpoints for interventions. Reanalysis with 3.3.1 Spiroware software of previously published data revealed a 10–15% decrease in LCI values [20,24]. Indeed, we also found that data recalculated with Spiroware 3.3.1 were lower for LCI and S_acin_ and higher for S_cond_; however, the differences were statistically significant only in children and adolescents during visits in stable condition. Kentgens and colleagues showed that in healthy children, the mean LCI 2.5% ± SD was slightly lower in updated Spiroware 3.3.1 when compared to the previous 3.2.1 version (6.3 ± 0.4 vs. 7.0 ± 0.5, respectively) [26]. In a study by Wyler and colleagues, the mean value ± SD of LCI in CF patients decreased from 9.99 ± 2.21 to 8.69 ± 1.8 using the corrected software [24]. In our study, the median LCI 2.5% in CF children in stable condition was also lower (9.35 in Spiroware 3.3.1 vs. 10.62 in 3.2.1, respectively). This is concordant with the fact that in people with already elevated LCI—such as cystic fibrosis patients with severe bronchopulmonary disease—the effect of erroneous software is greater [24].

Robinson and colleagues reanalyzed several datasets from already published studies including the one investigating changes in LCI during the episodes of clinical symptoms [27,28]. They found that the percent LCI change from stable baseline in school-aged children expressed as mean (95%CI) decreased from 8.9 (6.5; 11.3)% to 6.4 (4.3; 8.4)% using Spiroware 3.3.1 and 3.2.1, respectively. In our study, we noted a decrease from 1.79 (−0.34, 3.41) (median IQR) to 1.46 (−0.7, 4.25) in a change of the LCI absolute values and a decrease from 20.84 (−2.33, 32.25) to 11.17 (−4.7, 38.15) in LCI relative change using Spiroware 3.3.1 and 3.2.1, respectively. This data cannot be directly compared as our criteria for pulmonary exacerbation were more strict than in Perrem’s study, who, in fact, called them acute respiratory events; however, a similar trend is clearly visible [28]. In Perrem’s reanalysis, the data correction did not influence the statistical significance of interventions and had a beneficial impact on within-subject as well as between-subject variability of LCI [28].

Frauchiger and colleagues reanalyzed with their own script the raw data obtained in several studies including 303 participants from previous and ongoing trials including healthy children and cystic fibrosis patients [21]. They found a similar trend in LCI changes measured with both Spiroware software during exacerbations, although the magnitude of changes was smaller with the corrected Spiroware 3.3.1 software. The authors suggest that based on the results of their studies, the threshold for interventions’ efficacy should be redefined.

We also investigated changes in other than LCI MBW parameters. S_cond_ and S_acin_ might be referred to as ventilation inhomogeneity components: S_cond_ estimating flow in the distant conducting airways and S_acin_ the abnormalities at the convection–diffusion front close to alveoli, responsible for the diffusion abnormalities [29]. The median S_cond_ values measured with Spiroware 3.3.1 software were higher and those of S_acin_ were lower both in patients in stable condition and during exacerbations. Only the difference between median S_cond_ values in stable patients reached statistical significance (Table 2). This might imply that the sensor crosstalk error influenced the accuracy of measurements of the patency of conductive airways affecting the total ventilation inhomogeneity marker more (LCI). In the Exhalyzer D device, the N_2_ concentration is not measured directly. It is calculated from O_2_ and CO_2_ concentrations measured directly by the respective sensors. Therefore, the errors in these measurements would result in N_2_ concentration error. Most of the N_2_ measurement error was due to the O_2_ sensor underestimating O_2_ concentrations with increasing CO_2_ concentrations [20]. Although the highest error in N_2_ concentration occurred at very high oxygen concentrations at the end of the test according to Wyler [20], to our surprise, this error was apparently not large enough to significantly influence the S_acin_ values or any values during exacerbations. Interestingly, when we compared the differences in MBW parameters between two stable visits (ΔS) and exacerbations preceded by a stable visit (ΔS), these were statistically significant for LCI measured using both software and S_acin_ measured using only Spiroware 3.3.1 software (Table 3). Thus, with Spiroware 3.3.1 software, S_acin_ might offer potential as an additional marker of exacerbation in CF patients.

The limitation of our study is its retrospective nature and the fact that the data were collected in one center. Our results would need validation in future multi-center studies.

## 5. Conclusions

Our study, similarly to the other few existing reports, showed that despite lower LCI values obtained with the corrected Spiroware 3.3.1 software, the diagnostic value in the prediction of pulmonary exacerbations is comparable. This study adds to the currently available knowledge and data confirming the clinical utility of the modified Spiroware 3.3.1 software. We showed that the change of the Exhalyzer software, despite the difference in the LCI cut-off values, resulted in similar predictive values in pulmonary exacerbations in people living with cystic fibrosis.

## Figures and Tables

**Table 1:** Patients characteristic.

Variable	Median (Range) or Number (%)
Number of patients	47
Male/female	23/24
BMI (median; range)	18.44 (14.3–28.7)
BMI z-score < −1.64 *	6 (12.8%)
F508del homozygous	22 (46.8%)
F508 heterozygous	19 (40.4%)
FEV1 z-score < −1.64 *	23 (48.9%)
FEV1 z-score (median; range)	−0.39 (−12.10–4.15)
LCI 2.5% (median; range)	9.29 (4.51–23.93)
LCI 2.5% z-score (median; range)	8.93 (−6.3–52.34)

* During at least one visit. Abbreviations: FEV1—Forced vital capacity in the first second; BMI—body mass index; LCI—Lung clearance index.

**Table 2:** LCI parameters in stable patients and during exacerbations were measured with Spiroware 3.2.1 software and Spiroware 3.3.1 software. Median (25%; 75%).

Parameter	Spiroware 3.2.1	Spiroware 3.3.1	*p*-Value *
LCI 2.5% stable	10.62 (8.165; 14.5)	9.35 (7.38; 13.21)	0.005
LCI 2.5% exacerbations	13.86 (10.95; 18.44)	12.96 (9.88; 16.57)	0.08
S_cond_ stable	0.055 (0.033; 0.077)	0.066 (0.039; 0.09)	0.024
S_cond_ exacerbation	0.059 (0.036; 0.079)	0.068 (0.049; 0.095)	0.09
S_acin_ stable	0.121 (0.033; 0.077)	0.111 (0.068; 0.224)	0.53
S_acin_ exacerbation	0.152 (0.036; 0.312)	0.103 (0.099; 0.302)	0.97

* *p*-value calculated using Mann–Whitney test. Abbreviations: LCI %predicted—lung clearance index percentage of predicted value; LCI—lung clearance index (absolute value); S_cond_—index of conductive ventilation heterogeneity (absolute value); S_acin_—index of acinar ventilation heterogeneity (absolute value).

**Table 3:** Change in lung function parameters measured with Spiroware 3.2.1 and 3.3.1 software during pulmonary exacerbations and stable visits.

Lung Function Parameter Δ		Pulmonary Exacerbations ΔPE	Stable ΔS	*p*-Value *
LCI 3.3.1	Median (25%, 75%)	1.79 (−0.34, 3.41)	0.24 (−0.85, 0.19)	0.0009
LCI 3.2.1	Median (25%, 75%)	1.46 (−0.7, 4.25)	0.18 (−1.09, 1.36)	0.0079
LCI relative change 3.3.1	Median (25%, 75%)	20.84 (−2.33, 32.25)	2.7 (−7.97, 13)	0.0015
LCI relative change 3.2.1	Median (25%, 75%)	11.17 (−4.7, 38.15)	1.68 (−11.08, 11.01)	0.0050
S_cond_ 3.3.1	Median (25%, 75%)	0.01 (−0.01, 0.04)	0 (−0.01, 0.02)	0.1641
S_cond_ 3.2.1	Median (25%, 75%)	0 (−0.02, 0.02)	0 (−0.01, 0.02)	0.7313
S_acin_ 3.3.1	Median (25%, 75%)	0.04 (0, 0.06)	0 (−0.04, 0.04)	0.0202
S_acin_ 3.2.1	Median (25%, 75%)	0.01 (−0.04, 0.06)	0 (−0.04, 0.04)	0.4096

* *p* value calculated using Mann–Whitney test. Abbreviations: LCI—lung clearance index (absolute value); S_cond_—index of conductive ventilation heterogeneity (absolute value); S_acin_—index of acinar ventilation heterogeneity (absolute value).

## Data Availability

The datasets used and/or analyzed during the current study are available from the corresponding author upon reasonable request.
